# Diagnosis and management of multiple paragangliomas of the head and neck

**DOI:** 10.1007/s00405-014-3126-z

**Published:** 2014-06-12

**Authors:** Anna Szymańska, Marcin Szymański, Elżbieta Czekajska-Chehab, Wiesław Gołąbek, Małgorzata Szczerbo-Trojanowska

**Affiliations:** 1 Department of Interventional Radiology and Neuroradiology, Medical University of Lublin, Jaczewskiego 8, 20-154 Lublin, Poland; 2 Department of Otolaryngology, Head and Neck Surgery, Medical University of Lublin, Jaczewskiego 8, 20-154 Lublin, Poland; 3 Department of General Radiology, Medical University of Lublin, Jaczewskiego 8, 20-154 Lublin, Poland; 4 Professor Emeritus, Department of Otolaryngology, Head and Neck Surgery, Medical University of Lublin, Jaczewskiego 8, 20-154 Lublin, Poland

**Keywords:** Imaging, Metachronous, Multiple tumors, Paraganglioma, Surgery, Synchronous

## Abstract

Paragangliomas (PGs) are slowly growing, usually benign neoplasms. The aim of the study was to analyze the incidence, diagnostic and therapeutic management of patients with multiple paragangliomas of the head and neck. A retrospective review of the records of 84 patients with head and neck PGs, diagnosed and treated in our institution was performed for the years 1983–2013 to identify patients with multiple tumors. Fourteen (16.6 %) patients developed multiple PGs, synchronous or metachronous, within 4–21 years of follow-up. Clinical data of these patients were reviewed to evaluate the diagnosis, location, stage and management strategy. There was a total number of 37 tumors in 14 patients. There were 20/37 (54.0 %) carotid PGs, 9/37 (24.3 %) jugular PGs and 8/37 (21.7 %) vagal PGs. Carotid PGs were observed in 12/14 (86 %) patients and in 8/14 (57 %) cases bilateral tumors occurred. Vagal PGs developed in 7/14 (50 %) patients and bilateral tumors were found in 1/14 (7 %) case. Jugular PGs occurred in 9/14 (64 %) patients. There were 30 synchronous tumors and seven metachronous PGs diagnosed 2–18 years after removal of the first tumor. Single metachronous mediastinal PG occurred. All patients had at least one tumor removed, with histopathological confirmation of the diagnosis. One patient had positive history of familial PGs. Carotid PGs are most common multiple paragangliomas. Radiological survey of the head and neck is required to detect multicentric tumors. Metachronous mediastinal and abdominal tumors may occur. Regular, prolonged follow-up is essential to identify metachronous PGs and possible postoperative gradual ICA occlusion.

## Introduction

Paragangliomas (PGs) are slowly growing, usually benign neoplasms, which arise from extra-adrenal paraganglionic tissue derived from neural crest cells [1, 2]. In the head and neck region, parasympathetic-associated PGs may be located in four primary sites: the carotid bifurcation (carotid PG), the jugular bulb (jugular PG), the tympanic plexus (tympanic PG) and the vagal ganglia (vagal PG). Other, less common sites of origin are the nose, larynx, parotid gland, orbit and thoracic inlet [3]. Most of the tumors in the head and neck belong to the group of nonchromaffin PGs, as they do not secrete catecholamines and do not show catecholamine by chromaffin staining [4, 5].

It is difficult to evaluate the incidence of paragangliomas due to their rarity. It is estimated that they represent 0.012 % of all human tumors and 0.6 % of head and neck neoplasms [1]. Familial occurrence of PGs is well recognized and caused by mutations in succinate dehydrogenase (SDH) subunit genes [6, 7]. These tumors may be associated with several inherited syndromes: multiple endocrine neoplasia (MEN), neurofibromatosis type 1 (NF1), von Hippel-Lindau syndrome (VHL), paraganglioma syndrome (PGS) [1, 3]. Clinically very important are multiple paragangliomas, which may develop in the sporadic form of the disease, but are more common in heredofamilial variety.

The aim of the study was to analyze the incidence, diagnostic and therapeutic management of patients with multiple paragangliomas of the head and neck.

## Materials and methods

Between 1983 and 2013, 84 patients with head and neck paragangliomas were diagnosed and treated in our tertiary referral university hospital. Fourteen (16.6 %) of these patients had two or more tumors, synchronous or metachronous within 4–21 years of follow**-**up.

Clinical data of these 14 patients with multiple PGs were reviewed to evaluate the diagnosis, location, stage and management strategy. The study was approved by the institutional ethical committee.

All patients with multiple paragangliomas had at least one tumor removed, with histopathological confirmation of the diagnosis. There were nine females and five males and the age of patients ranged from 14 to 62 years.

In total MRI was performed in 13 patients, CT in 10 patients and ultrasound study (US) in 12 patients. Superselective transarterial embolization of tumor vessels was performed only in patients with jugular PG, 2 or 3 days before surgery. Jugular PGs were classified according to Fisch staging system [8] (Table 1).

**Table 1:** Classification of temporal paragangliomas by Fisch et al. [8]

Stage	Description
Class A	Tumors arise along the tympanic plexus on the promontory of the middle ear. Minimal erosion of the promontory may occur
Class B	Tumors originate in the canalis tympanicus of the hypotympanum and invade the middle ear and mastoid. The carotid foramen and canal are intact. Tumors invade the bone of the hypotympanum, but the cortical bone over jugular bulb is intact
Class C	Tumors originate in the dome of the jugular bulb and destroy overlying cortical bone and may spread inferior, posterior, superior, lateral and medial
C1	Erosion of the carotid foramen, without invasion of the carotid artery
C2	Destruction of the vertical carotid canal between the carotid foramen and the carotid bend
C3	Involvement of the horizontal portion of the carotid artery, without the foramen lacerum
C4	Involvement of the foramen lacerum and along the carotid artery of the cavernous sinus
Class D	Class D indicates the intracranial extension of the tumor. Intracranial extension may be extradural (De) or intradural (Di)
De 1	Tumors displace the posterior fossa dura <2 cm
De 2	Tumors displace the posterior fossa dura >2 cm. The tentorium may be pushed superiorly
Di 1	Intradural extension <2 cm. No involvement of the pontomedullary brainstem
Di 2	Intradural extension >2 cm, attached to the vascular and neural structures of the brainstem
Di 3	Neurosurgically unresectable tumors

## Results

### Symptoms

Symptoms of jugular PGs included ipsilateral hearing loss and tinnitus. Evaluation of the tympanic membrane revealed bulging and/or pulsating polyp. Carotid PGs on clinical examination presented as palpable, non tender lateral neck masses. Vagal PGs manifested with a palpable, painless mass in the upper neck. In advanced tumors medial protrusion into the oropharynx was observed. Three patients presented with facial nerve palsy and four patients with lower cranial nerve palsy (IX–XII). In 8 patients 13 small synchronous tumors were asymptomatic, and discovered with CT, MRI or ultrasound.

### Imaging

Carotid PGs were diagnosed on the basis of US, CT and/or MRI. US depicted carotid PGs in all patients evaluated with this modality. All tumors were located within the carotid bifurcation, caused splaying of the internal (ICA) and external carotid arteries (ECA) and had multiple blood vessels with arterial blood flow of low-resistance pattern on Doppler study. US was not sufficient for reliable diagnosis of vagal PGs.

**Fig. 1:**
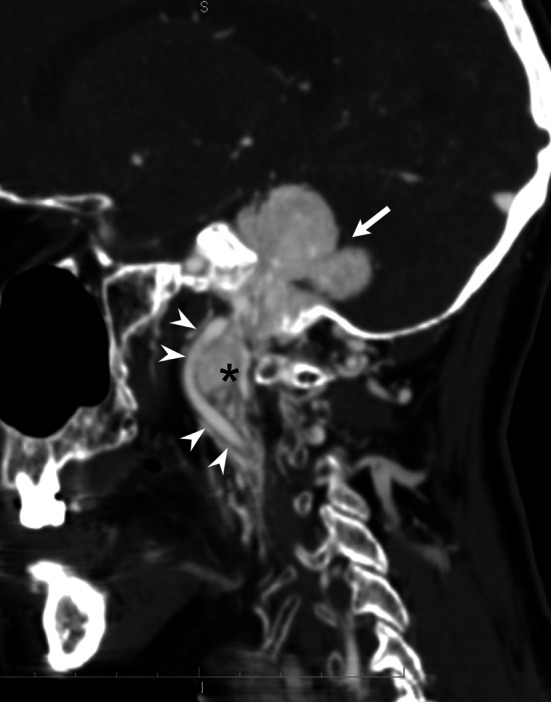
Contrast-enhanced sagital CT reconstruction well demonstrates double PG on one side of the head and neck: advanced intradural jugular PG (*arrow*) and vagal PG (*asterisk*) with anterior displacement of the internal carotid artery (*arrowheads*)

**Fig. 2:**
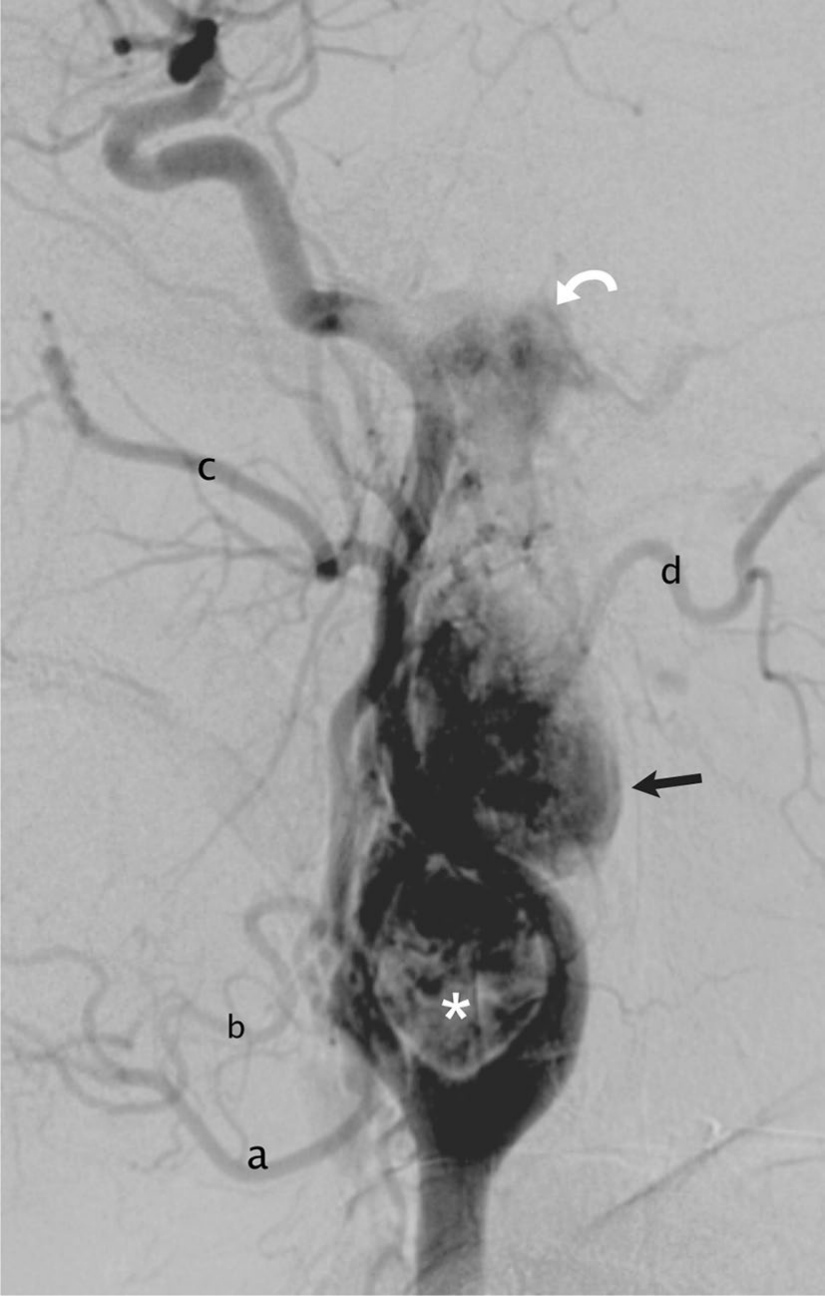
Common carotid angiography, lateral view, shows three synchronous hypervascular tumors: jugular PG (*curved arrow*), vagal PG (*arrow*) and carotid PG (*asterisk*). Branches of the ECA: **a** facial artery, **b** lingual artery, **c** internal maxillary artery, **d** occipital artery

All jugular and vagal PGs were diagnosed on the basis of high resolution CT and MRI (Fig. 1). In all jugular PGs, CT scans showed irregular widening of the jugular foramen with cortex erosion and permeative destruction of the surrounding bone. Vagal PGs did not cause erosion or widening of the jugular foramen. In all PGs, CT scans demonstrated solid tumors with intensive, homogeneous contrast enhancement. On MRI all PGs showed intermediate to high signal intensity on T2-weighted images, low to intermediate signal intensity on T1-weighted images and intensive contrast enhancement. Intratumoral flow voids were visible in 11 PGs. Angiography in all cases demonstrated extremely vascular tumors with intense blush and apparent arteriovenous shunts in five cases (Fig. 2). All tumors were supplied by the ECA and in two patients by the branches of the ICA.

### Tumor characteristics

There was a total number of 37 head and neck tumors in 14 patients. There were 20/37 (54.0 %) carotid PGs, 9/37 (24.3 %) jugular PGs and 8/37 (21.7 %) vagal PGs. Carotid PGs were observed in 12/14 (86 %) patients and in 8/14 (57 %) cases bilateral tumors occurred. Vagal PGs developed in 7/14 (50 %) patients and bilateral tumors were found in 1/14 (7 %) case. Jugular PGs occurred in 9/14 (64 %) patients, no bilateral tumors were observed.

There were 30 synchronous tumors and seven metachronous head and neck PGs diagnosed 2–18 years after removal of the first tumor. One patient in the group (No. 7, MM) had 5 PGs: carotid, vagal and jugular tumors on one side, a carotid tumor on the opposite side and a metachronous aorticopulmonary PG. One patient (No. 8, JA) developed three synchronous and one metachronous PG 5 years after resection of pheochromocytoma.

One patient (No. 8, JA) had positive history of familial PGs. No secretory activity was found.

### Treatment strategy

All patients with multiple PGs underwent surgical treatment. Symptomatic tumors or tumors with radiologically confirmed progression were removed. Multiple PGs on one side of the head and neck were removed in single-stage. In small, symptomless tumors without evidence of progression a wait and scan policy was employed. In bilateral PGs after extirpation of one side tumor the ICA patency and cranial nerve functions were monitored. Only in case of normal clinical and radiological findings the second stage operation of the contralateral tumor was considered. Otherwise conservative a wait and scan policy was adopted.

### Follow-up

One patient (No. 2, RA) with jugular, vagal and carotid PGs on one side of the head and neck died due to the ICA rupture 5 days after single-stage removal of the three tumors. Patient No. 5 (DA) with advanced temporal paraganglioma (Fisch C1Di2) and severe neurological deficits underwent nonradical surgery and died 3 years after the operation due to progression of the disease. Another patient (No. 6, DM) died 4 years after surgery of advanced jugular and vagal PGs (Fisch C4Di1) because of breast cancer. In other patients control of the disease has been achieved up to 21 years follow-up, e.g. patients are without clinical and radiological signs of progression of the disease. Two patients with large bilateral carotid PGs (No. 11 KJ, No. 12 WM) after removal of one tumor and repair of the carotid injury developed gradual occlusion of the ipsilateral ICA, without neurological deficits.

Detailed clinical data of the patients with multiple PGs are depicted in Table 2.

**Table 2:** Clinical data of 14 patients with multiple paragangliomas

No	Initials, age, sex	Cranial nerve palsy at presentation	Right PGs			Left PGs			Cranial nerve palsy after first operation	Time to metachronic tumor presentation (years)	Follow-up since first presentation (years)	Other pathology
			Jugular	Vagal	Carotid	Jugular	Vagal	Carotid				
1	DW, 14, M	VII, IX, X, XI XII			*SUR (II)*	SUR ^a^ [C2De1]	SUR ^a^		VII, IX, X, XI XII	18	20	
2	RA, 54, F	VII, IX, X, XI, XII	SUR [C1De1]	SUR	SUR (II)				VII, IX, X, XI, XII		–	Died on the 5th postoperative day due to carotid rupture
3	BM, 34, F		SUR [C2]		*OBS*			*SUR (II)*		5	17	
4	LE, 14,F		SUR [C1]					OBS	VII- HB2		10	
5	DA, 62, F	VII, IX, X, XI, XII	SUR [C1Di2]	SUR					VII, IX, X, XI, XII		–	Died 3 years after surgey
6	DM, 61, F	X, XI, XII	SUR [C4De1]	SUR					X, XI, XII		–	Died 4 years later due to advanced breast cancer
7	MM, 39, F				OBS	RT [C2]	SUR	SUR (II)		2	5	**Metachronic** inoperable mediastinal PG treated with RT
8	JA, 40, M		SUR ^a^ [C2]	SUR ^a^	*OBS*			SUR (II)		2	4	Suprarenal tumor resected 5 years before ENT presentation
9	SG, 50, F		*OBS*		SUR (III)					17	20	
10	MG, 36, F			OBS	SUR (II)		OBS	OBS			14	
11	KJ, 56, M				SUR (III)			OBS			12	Gradual right internal carotid occlusion after surgery
12	WM,44,M				OBS			SUR (III)	X, XI		5	Gradual left internal carotid occlusion after surgery
13	LA, 35, F				SUR (II)			*SUR (II)*		8	14	
14	GR, 49, M				SUR (III)			*OBS*	X, XI	15	21	

Stage according to Fisch [] classification for temporal paraganglioma, and Shamblin () for carotid paraganglioma in brackets

Management of PGs: SUR, surgery; RT, radiotherapy; OBS, observation; SUR means tumors resected in a single stage; *SUR* means metachronic tumor

^a^Point tumors treated first

## Discussion

Multiple paragangliomas may occur synchronously or metachronously. Multicentric tumors occur in 10–20 % of all head and neck paragangliomas [2, 9–11]. However, reports of much higher incidence of multiple tumors, like 40 % for sporadic form and 80 % for familial variety, can be found in the literature [5, 12, 13]. Bilateral tumors have been reported in 4.4 % of sporadic and 31.8 % of familial cases [1]. In the total number of 84 paraganglioma patients treated in our institution, 16.6 % of patients had multiple tumors and vast majority of them were synchronous. It is noteworthy that some multicentric PGs are asymptomatic at presentation of the disease. Gardner et al. [5] discovered 13 unsuspected paragangliomas in the total number of 23 tumors diagnosed. There were 13 asymptomatic PGs in our group of 14 patients with 37 multiple paragangliomas. The most common multiple paragangliomas are carotid body tumors, which constitute ~10 % of sporadic cases and 25–33 % of familial cases [2, 13]. In our study carotid PGs represented more than a half of all tumors and 57 % of patients had bilateral carotid PGs. According to Van Baars et al. [14], at least one carotid body tumor was found in every patient with multiple PGs. In two patients (No. 5 and No. 6) from our group no carotid body tumors were detected. However, in two other patients (No. 1 and No. 3) carotid PGs developed as metachronous tumors 5 and 18 years after resection of temporal bone and vagal tumors, respectively. Multiple vagal PGs represent about 17 % of all cases [2]. Bilaterality is uncommon, with eight cases of bilateral vagal PG reported in the literature [2].

The familial occurrence of paragangliomas has been well recognized and accounts for 10–40 % of cases [9, 15]. While no sex correlation exists, the familial form of the disease may become clinically apparent at a younger age [10]. In our study only one patient had a positive family history of PG and he was 40 years old at the initial presentation of the disease. The youngest patients in this study were 14 years old at the time of diagnosis. In previous reports the youngest patients identified with this disease were 16 years old [13]. As the morbidity of surgical treatment of PGs increases with their size, it is suggested that patients at risk should be screened by physical examination, urinary catecholamine analysis and magnetic resonance imaging of the head and neck every 1 or 2 years beginning with 14–16 years of age to detect tumors at an early stage [13, 15].

**Fig. 3:**
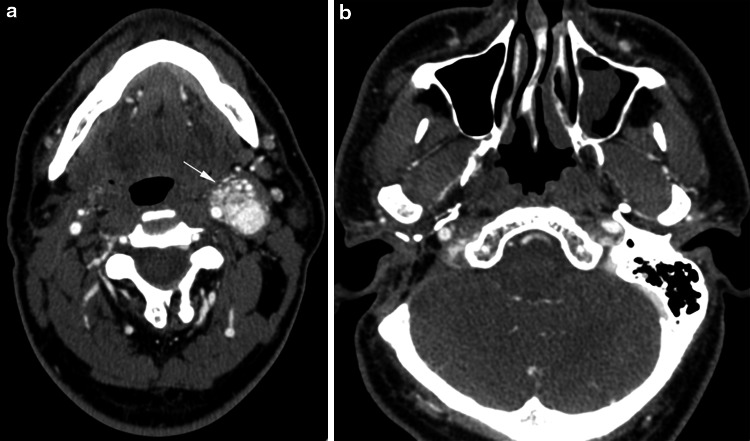
Axial post-contrast CT scans demonstrate large carotid PG (**a**) (*arrow*) in a patient after single-stage infratemporal removal of contralateral, synchronic jugular and vagal PGs (**b**)

Tumor growth in PGs is difficult to predict. Most PGs in the head and neck grow very slowly and remain asymptomatic for years. However, there are cases of rapid tumor growth and this is particularly noticeable in some patients with familial form of the disease [3, 15]. We observed rapid progression of small synchronous carotid PG in the patient (No. 8, JA) after previous surgical removal of jugular and vagal tumor (Fig. 3a, b). This was the only patient in our group with familial history of paraganglioma.

Although the majority of paragangliomas and pheochromocytomas are benign, both the sporadic and familial forms of PGs have the potential to be malignant. The criteria of malignancy based on histopathology are not well defined and the only accepted evidence of malignancy is metastasis [15]. It is estimated that <5 % of all PGs are malignant [10, 15]. Malignancy is most common in vagal PGs [11]. In his study, Lee et al. [16] reviewed the records of the National Cancer Data Base from 1985 to 1996 and found 59 (0.07 %) malignant PGs. In this group 31 % of patients had distant metastases and about 69 % presented with regional lymph node metastases, which had much better prognosis than the systemic ones. Neumann et al. [17] found 33 malignant tumors in an international study comprising 598 patients. In the present series of multiple paragangliomas no malignant tumors occurred and there was only one case of malignant vagal PG in the whole group of 84 paraganglioma patients treated at our institution.

**Fig. 4:**
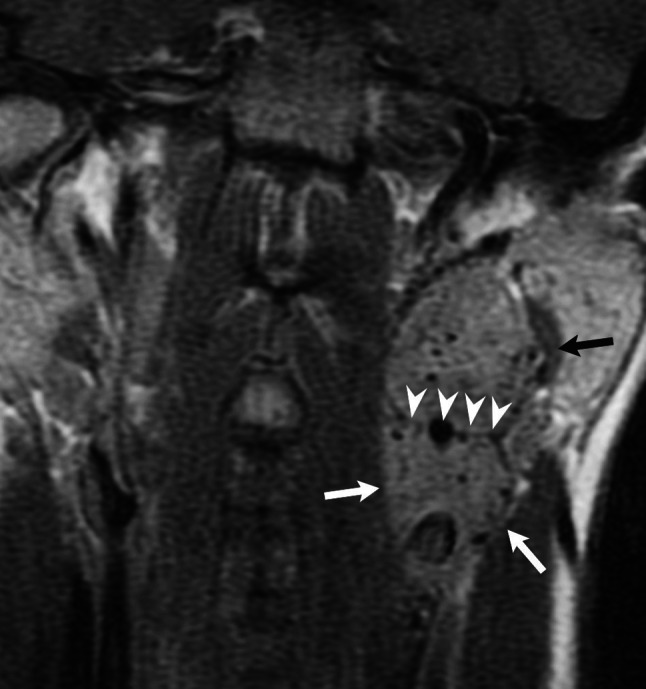
Coronal T1-weighted MR image after application of contrast medium shows thin border (*arrowheads*) between two ipsilateral tumors: vagal PG (*black arrow*) and carotid PG (*white arrows*)

At the onset of the disease radiological imaging has two main goals: aid the clinical diagnosis and depict multiple tumors, which may occur synchronously or metachronously. Ultrasound has an established role in the management of neck tumors. Doppler US should be the first imaging procedure employed in patients with suspected carotid PG, as it demonstrates abundant vascularity of PGs and facilitates differentiating them from other solid, non hypervascular neck masses or enlarged vascular spaces, such as carotid artery aneurysm [2]. Identification of carotid PG implies complete US imaging of the neck and both bifurcations, as bilateral lesions may occur and synchronous vagal PG may also be present. However, US usually cannot delineate vagal PGs, as they are located farther cephalad in the neck, as well as the superior aspect of large carotid PGs. In such cases contrast-enhanced MRI should be performed to locate the tumor accurately (Fig. 4). US may also be used to assess carotid PG growth in follow-up of patients, when wait and scan policy was chosen. In patients with suspected jugular PG the initial imaging modality should be contrast-enhanced CT of the brain and skull base to delineate the tumor and a high resolution CT scan of the temporal bone to demonstrate typical for PGs bone changes, e.g. enlargement of the jugular foramen with erosion of its cortical margins [9, 18]. With better tissue characterization MRI may be helpful in further differential diagnosis and provides precise visualization of intracranial involvement in advanced tumors. Due to improved soft-tissue definition, this modality has been used as a non-invasive screening technique to detect subclinical synchronous neck PGs. Angiography demonstrates typical for paragangliomas vascular blush and confirms the clinical diagnosis. It reliably rules out the presence of multiple synchronous tumors. For this purpose the diagnostic algorithm should include, apart from selective catheterization of ECA and ICA, contrast injection to both common carotid arteries to visualize potential small carotid PGs. Knowledge of the integrity of the contralateral cerebral circulation is essential to determine the management of the ipsilateral ICA during surgery and due to the risk of its gradual occlusion after tumor removal, which was observed in two of our patients. Some authors perform angiographic balloon test occlusion with transcranial Doppler monitoring to predict correctly hemodynamic outcome of potential sacrifice of the ICA [19]. However, in multiple paragangliomas, especially bilateral tumors, the decision: a sacrifice of the ICA versus subtotal resection should be thoroughly considered and should depend on the treatment planning of the opposite side tumors. Angiography is no longer mandatory in diagnostic management, but is a last step in preoperative evaluation for selection of patients, who may benefit from preoperative embolization [3, 9]. We performed carotid angiography with embolization of tumor vessels only before surgery of jugular PGs and synchronic ipsilateral vagal PGs. Due to expanding capabilities of CT and MR angiography, non-invasive visualization of tumor vascular nature, arterial feeding branches and venous compression or invasion is possible [3].

Treatment options for head and neck paragangliomas include: surgery, conventional radiotherapy, stereotactic radiotherapy and stereotactic radiosurgery (for temporal bone PGs), permanent embolization and combination of these treatment modalities. The choice of treatment method depends on many factors, such as the age and general health of the patient, location and extensions of the tumor, tumor size and growth rate, carotid artery involvement, cranial nerve deficits and potential risks connected with particular treatment modality.

Adequate tumor staging is important in discussing the optimal treatment modality, assessment of potential risks of surgery and selection of the surgical approach. Based on tumor location and extension, staging system introduced by Fisch et al. [8] is widely used for evaluation of temporal bone PGs. Shamblin et al. [20] introduced classification of carotid PGs reflecting the involvement of the carotid artery and difficulty of tumor resection.

Due to very slow progression of the disease, in some cases a wait and scan policy may be employed [3, 15]. Complete surgical resection is considered a treatment of choice for the majority of patients with carotid paragangliomas [3, 5]. The optimal treatment for jugular and vagal PGs is more controversial. Potential risks of surgery for these tumors are permanent cranial nerve deficits resulting in vocal cord palsy and aspiration or pneumonia, as well as facial palsy for jugular PGs. As patients with preoperative lower cranial nerve palsy have time to compensate, they are better candidates for surgery, than those with normal nerves function. According to a review of over one thousand operated jugular PGs, 1.6 % of patients died due to postoperative complications. In a group of 226 vagal PGs perioperative mortality rate was 1.3 % [15]. To avoid lower cranial nerve palsy, incomplete resection of large tumors in the jugular foramen with postoperative radiotherapy may be justified. Sivalingam et al. [21] apply two stage removal of tumors with intradural extension, e.g. neurosurgical and infratemporal fossa approach. In case of advanced intracranial invasion, class Di3 tumors, palliative radiation therapy is advocated [3].

Conventional radiotherapy and stereotactic radiotherapy have been initially used in cases of unresectable tumors, patients with poor general condition, or those who refused surgery. However, serious complications have been reported after irradiation of jugular PGs, including temporal bone osteomyelitis, temporal bone necrosis and risk of radiation induced malignancies [3, 22]. Currently the role of radiotherapy is increasing, with special interest in stereotactic radiotherapy and radiosurgery, which spare adjacent normal tissues [23, 24]. Suarez et al. [15] reviewed 75 studies and compared the results of treatment of 1,084 patients with jugular PGs and of 226 patients with vagal PGs. They concluded that in patients with jugular PGs radiotherapy offers similar chance of tumor control with lower risk of morbidity in comparison with surgery. The authors suggest surgery only for patients with small jugular PG and radiotherapy for the rest. Surgery for vagal PGs should be reserved for patients that already have vagal nerve palsy.

Management of multiple paragangliomas is even more difficult. In management of such a rare and challenging disease, centralization of treatment would enable experienced institutions to specialize in this type of surgery and to reduce the mortality and morbidity. Special consideration is required in planning the initial and potential future treatment. For patients with bilateral vagal and jugular tumors, every effort must be made to preserve vagal function and hearing at least on one side [15, 18]. Van der Mey et al. [25] indicate that a wait and scan policy should be considered for such patients. Surgery is advisable only in serious progressive cranial nerve deficits or life-threatening intracranial growth. As recognized in two of our patients after carotid body tumor resection, ipsilateral carotid diameter may decrease with time leading to ICA occlusion, which also has an impact on further treatment planning. In bilateral tumors, Myssiorek et al. [1] suggest removal of small carotid PG prior to resection of contralateral large carotid or any vagal PG. If there is already a vagal nerve palsy due to the tumor or surgery, a contralateral carotid PG should be resected only in case of documented rapid growth. In multicentric disease, Boedeker [3] and Velegrakis et al. [26] suggest removing the largest tumor. The remaining PGs can be observed or radiated depending on the function of the cranial nerves. Sobol et al. [10] advise one step surgery for patients with unilateral multiple PGs. Similar approach was applied in our patients.

The follow-up is particularly important in management of paragangliomas. In case of a wait and scan strategy potential tumor growth may be monitored by means of MR imaging and the intervals between subsequent MR studies should depend on the clinical situation of each patient. The suggested follow-up interval in clinically stable patients is approximately 2 years. Progressive neurological deficits and deterioration require a shorter imaging intervals [18].

Postoperative follow-up is based on MRI and involves surveillance of the whole head and neck to detect multicentric tumors. To improve the visualization of recurrent paraganglioma in the temporal bone after surgery the use of fat-saturated contrast-enhanced T1-weighted images is very important. During first 5 years after operation 6 months intervals are recommended. Thereafter, they may be stretched to 3 to 5 years [3].

In postoperative follow-up of patients after resection of head and neck paraganglioma the possibility of metachronous tumors should be considered. We have detected seven metachronous PGs in six patients 2–18 years after the first presentation of the disease. Apart from head and neck tumors, in one patient metachronic mediastinal PG developed. This confirms the importance of prolonged, regular follow-up of PG patients, which should include clinical examination and radiological imaging. Abdominal extra-adrenal paragangliomas and pheochromocytomas usually hypersecrete catecholamines and produce certain clinical symptoms, therefore, physical examination and urinary catecholamine analysis are useful screening methods for these tumors. In patients at risk abdominal ultrasound and MRI may be used as screening tools. This is particularly valuable in patients with hereditary paragangliomas, as multicentricity is more frequent in this form of the disease. Functional imaging like radionuclide scintigraphy and positive emission tomography (PET) allow screening of the whole body to detect multiple tumors or metastatic disease [27].

## Conclusions

Carotid body tumors are most common multiple PGs and most common bilateral tumors. In patients with head and neck PGs diagnostic imaging should always involve surveillance of the whole head and neck to detect possible synchronous asymptomatic multicentric tumors. As multiple paragangliomas may develop many years after the presentation of the first tumor, regular, prolonged clinical and radiological follow-up of these patients is required. Apart from head and neck, other locations of metachronous tumors should be also considered. Follow-up imaging should include evaluation of the ICA patency, as gradual occlusion of the artery may occur and influence further treatment planning. Management of multiple head and neck paragangliomas should take into account age and general condition of patients, size and location of tumors, cranial nerve function and possible synchronous tumors.
